# Volar locking plates not touching the flexor pollicis longus tendon appear as prominences on radiographs: a cadaver study

**DOI:** 10.1186/s10195-019-0536-0

**Published:** 2019-08-20

**Authors:** Kotaro Sato, Yuki Kikuchi, Yoshikuni Mimata, Kenya Murakami, Gaku Takahashi, Minoru Doita

**Affiliations:** 1 Department of Orthopaedic Surgery, Iwate Medical University, 19-1 Uchimaru, Morioka, Iwate 020-8505 Japan; 2 Department of Critical Care Medicine, Iwate Medical University, 19-1 Uchimaru, Morioka, Iwate 020-8505 Japan

**Keywords:** Anatomical study, Distal radius fracture, Flexor pollicis longus, Tendon rupture, Volar locking plate

## Abstract

**Background:**

Plate protrusion is a risk factor for flexor pollicis longus (FPL) rupture following volar locking plate (VLP) surgery. However, plate prominence on follow-up radiographs is common. We hypothesised that a VLP that does not touch the FPL tendon can appear as a plate prominence projected over the volar ridge on lateral radiographs.

**Materials and methods:**

We studied six current designs of widely used plates in formalin-fixed cadavers. Each plate was placed in six cadavers. We analysed 36 different plate–cadaver combinations. The main aim of plate fixation was to position the plate in the most distal position without FPL tendon contact. Radiographs were obtained using fluoroscopy. We evaluated plate prominence from the volar ridge according to the Soong grading system.

**Results:**

Soong grades 0 (plate did not extend beyond volar ridge), 1 (plate protruded beyond volar ridge) and 2 (plate directly on or located beyond the volar ridge) were observed in 23 (63.9%), 9 (25.0%) and 4 (11.1%) cadavers, respectively. VariAx, DVR and VALCP showed grade 1 prominence, whereas Acu-Loc2, HYBRIX and MODE showed grade 2 prominence.

**Conclusions:**

Implant protrusion was observed in 36% of plate–cadaver combinations, even if the plate did not touch the FPL. Estimating the risk of FPL rupture using lateral radiographs alone is likely insufficient. Our findings can be applied to accurately identify the presence of implant prominence following VLP surgery.

## Introduction

The volar locking plate (VLP) system provides stable internal fixation to allow early rehabilitation and has been widely used for patients with distal radius fracture (DRF) [1]. Flexor pollicis longus (FPL) rupture is a serious complication that may occur when using the VLP system [2, 3]. Azzi et al. reported that the incidence of tendon rupture was 1.5% for volar plates and 1.7% for dorsal plates [4]. FPL was the most commonly ruptured tendon, with the flexor digitorum profundus to the index finger being the second most common [5]. Previous studies have tried to identify risk factors for tendon rupture associated with VLP surgery [6–8].

The watershed line was first described by Orbey and Touhami and defined as a transverse ridge bordering the pronator fossa distally [9]. This bony prominence is known as the distal limit of the VLP. Imatani et al. reported that the medial side of the volar ridge was a good landmark for the distal limit of the safe area [10]. Many reports have suggested that projection of the plate over the volar ridge may cause FPL tendon rupture [2, 6, 7]. However, prominence of the plate after VLP surgery is commonly seen in follow-up radiographs, and not all protrusions cause FPL rupture [11, 12]. It may thus be considered that plate protrusion observed on radiographs does not always indicate direct contact with the FPL tendon.

Appropriate indications for hardware removal have not been determined [13]. If patients complain of pain or crepitation when moving the thumb on routine medical examination, implant removal is recommended [12]. Based on radiological findings, surgeons consider implant removal when lateral radiographs show prominence of the plate beyond the volar ridge. However, mandatory removal of all protruded implants would be burdensome and costly to patients.

The aim of this study is to evaluate lateral radiographs of cadaver wrists with various VLPs placed in the most distal position without FPL tendon contact. We hypothesised that a VLP that does not touch the FPL tendon may appear as a plate prominence projecting over the volar ridge on lateral radiographs.

## Materials and methods

### Dissection, plate fixation and radiographs

**Fig. 1:**
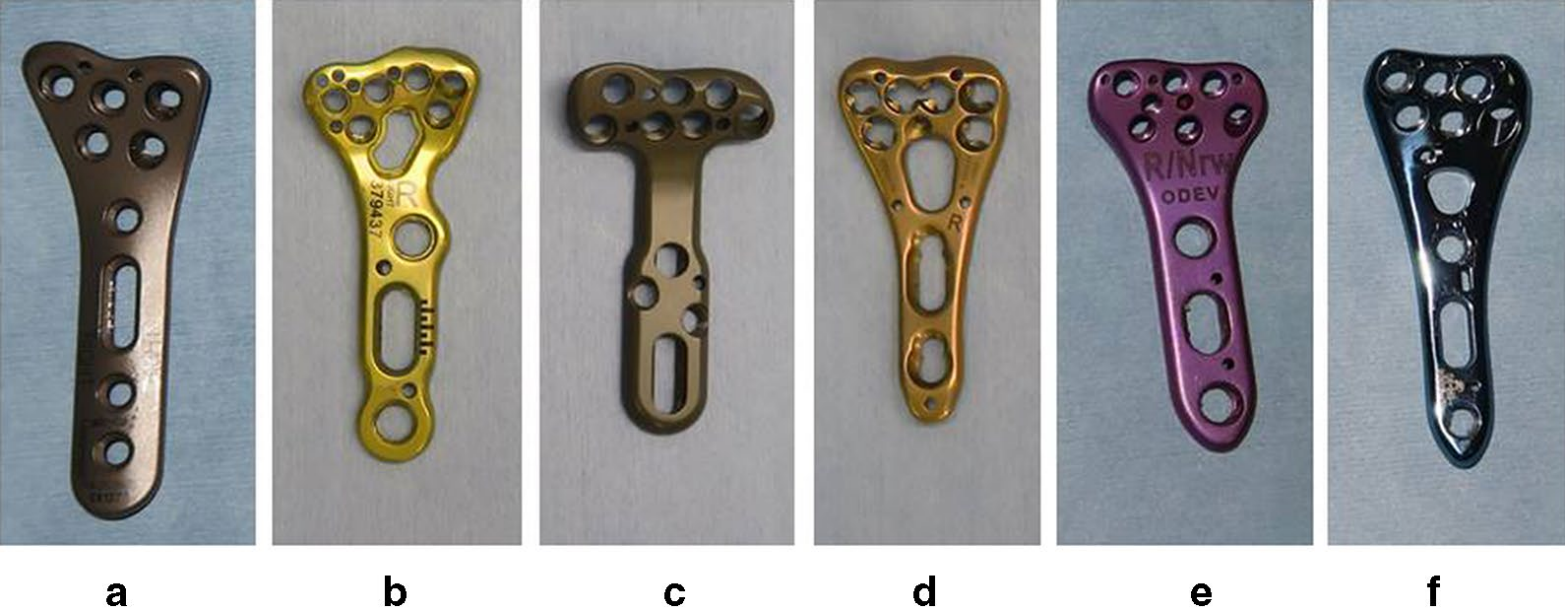
Six current designs of widely used plates: **a** VariAx (Stryker), **b** Acu-Loc2 (AcuMed), **c** DVR (Zimmer Biomet), **d** VALCP (Depuy Synthes), **e** MODE (JAPAN MEDICAL DINAMIC MARKETING INC) and **f** HYBRIX (Mizuho)

We studied six current designs of widely used plates, viz. the VariAx distal radius locking system (VariAx, Stryker, Kalamazoo, MI), Acu-Loc2 Proximal VDR plate (Acu-Loc2; Acumed, Hillsboro, OR), DVR anatomic plate (DVR; Zimmer Biomet, Warsaw, IN), variable-angle LCP two-column volar distal radius plate 2.4 (VALCP; Depuy Synthes, West Chester, PA), MODE (MODE; JAPAN MEDICAL DINAMIC MARKETING INC, MDM, Tokyo) and HYBRIX (HYBRIX; Mizuho, Tokyo, Japan) (Fig. 1). Each plate has an anatomical precontoured design, and manufacturers recommend that its position should not be beyond the watershed line. Nine formalin-fixed elbow-to-hand cadavers (seven male, two female; age 67–89 years) without severe degenerative or traumatic changes were used. The mean width of the distal radius was 31 mm (range 27–34).

**Fig. 2:**
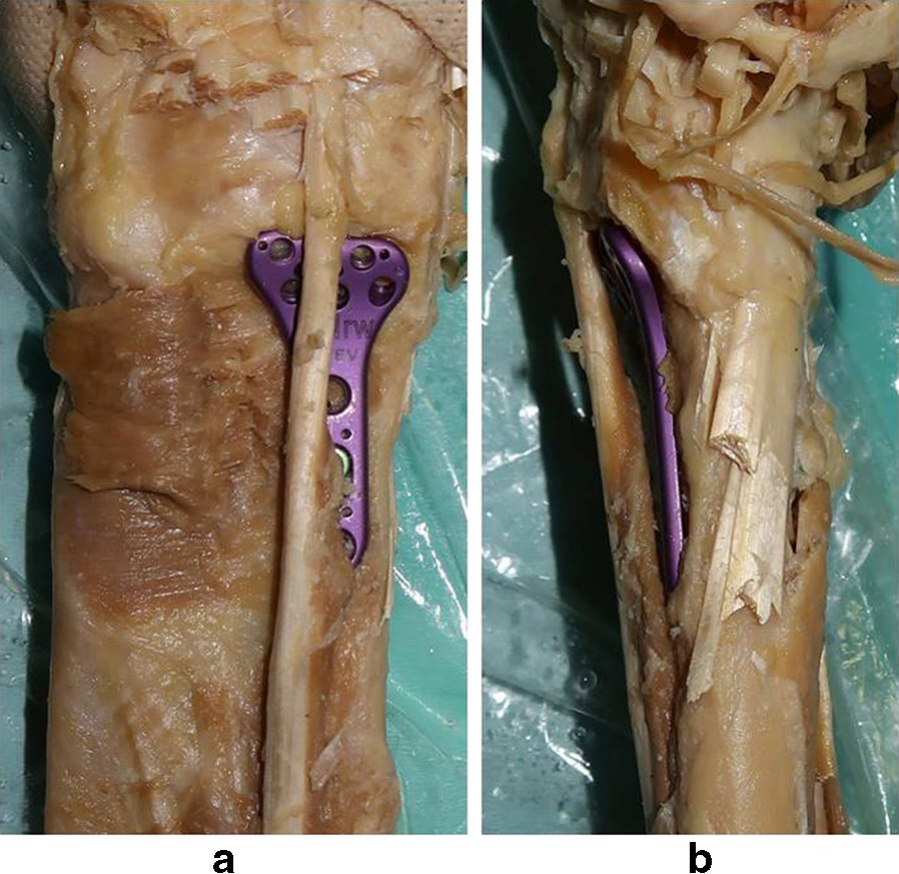
**a** Plate placement viewed from the front. Flexors except the flexor pollicis longus (FPL) are removed. **b** Plate placement viewed from the lateral side. The plate is fixed to the cadaver in the most distal position without FPL tendon contact

Dissection began by removing the skin and soft subcutaneous tissue on the forearm to expose the flexor muscle group. The FPL muscle origin was maintained in its anatomical position during the dissection. To confirm the relationship between the FPL and plate prominence, all flexor muscles and pronator quadrates were cut, except the FPL. This process was mandatory, because if other flexors were retained, it would not be possible to confirm the contact between the FPL tendon and plate. Other soft tissues, such as joint capsules, ligaments and fat tissue around the wrist joint, were preserved. We randomly selected six from nine cadavers and allocated them to each plate, so that each plate was placed in six different cadavers; thus, there were 36 different plate–cadaver combinations. The priority during plate fixation was to position the plate in the most distal position without FPL tendon contact with best fitting to the radial and ulnar positions (Fig. 2a, b). For each of the six plates, the smallest size was selected to exclude selection bias. Such size is commonly used in clinical practice for Japanese patients. All plate fixations were performed by one hand surgeon under direct vision. First, the plates were fixed with a cortical screw in the oval hole sufficiently distally to make contact with the FPL tendon, then the plate was slightly moved sufficiently proximally to relieve the contact during 30° wrist extension [14]. Finally, the plates were fixed with a locking screw. Thereafter, another orthopaedic surgeon confirmed that the plate was not in contact with the FPL tendon and that there was no room to position the plate more distally. To minimise the potential bias related to the order of plate fixation, each plate was randomly assigned to the cadavers. The plate was fixed to the radial shaft through the same cortical screw hole, if fixation was maintained. When the plate could not obtain rigid fixation because of screw loosening, another hole was drilled and secure fixation achieved. If necessary, two or more cortical screws were used. Lateral radiographs were obtained using fluoroscopy by confirming the projection of the pisiform over the distal portion of the scaphoid according to the methods of Soong et al. [7]. The radiograph was taken several times (one to four, mean 2.2), and the radiograph showing the greatest plate projection was used for analysis. A posteroanterior radiograph was also obtained to confirm the coronal plane of the distal radius and implant.

### Measurements

**Fig. 3:**
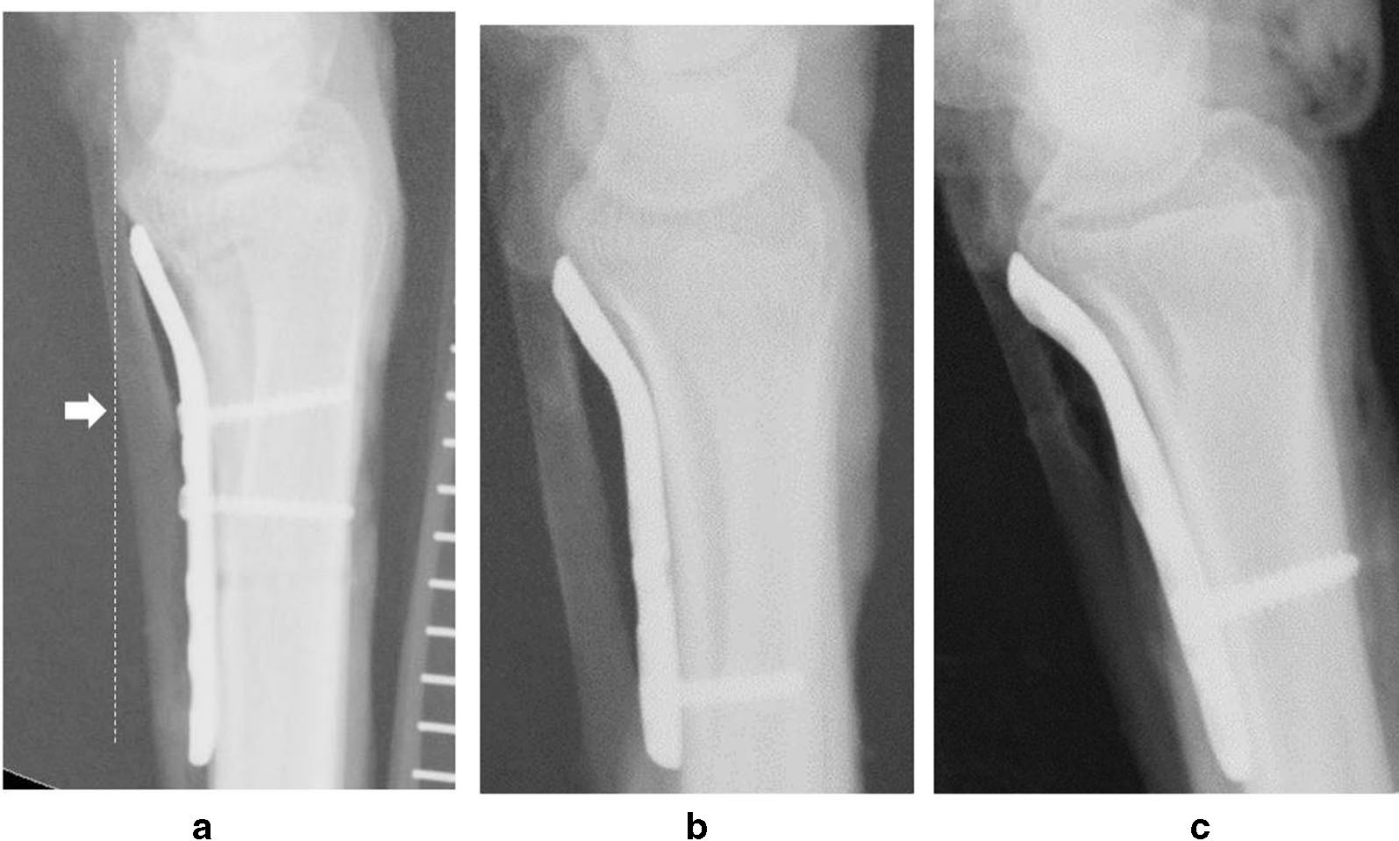
**a** Soong grade 0. VariAx is used. White arrow indicates critical line. Flexor pollicis longus is visualised using contrast agent. **b** Soong grade 1. DVR is used. **c** Soong grade 2. Acu-Loc2 is used

**Fig. 4:**
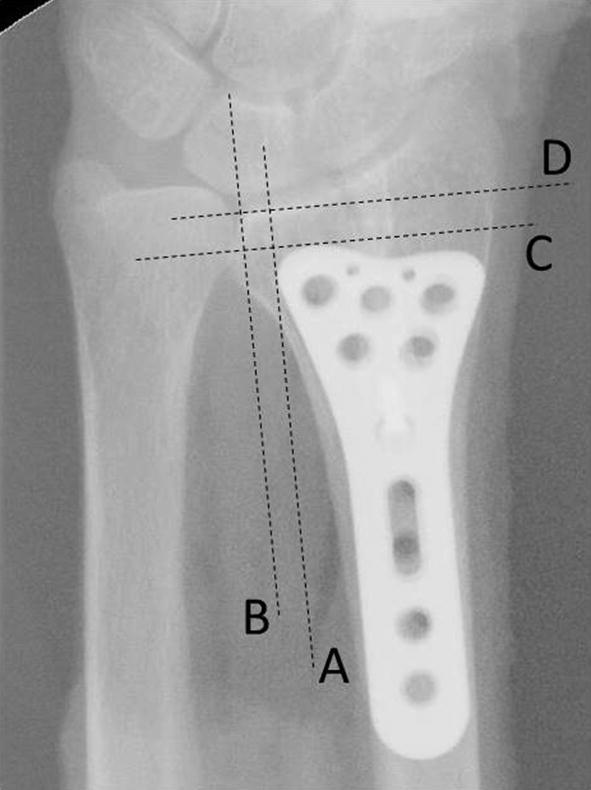
Lines A, B: A line is drawn parallel to the radial shaft over the plate end and radius ulnar border. The distance between points A and B indicates plate-to-radius ulnar border distance (PRU distance). Lines C, D: A line is drawn perpendicular to the radial shaft over the plate end and distal radius. The distance between points C and D indicates plate-to-distal radius distance (PDR distance)

We evaluated plate prominence from the volar critical line according to the Soong grading system [7]. The critical line was drawn parallel to the volar cortex of the radial shaft touching the most volar tip (Fig. 3a). Soong grade was defined as follows: plates that did not extend the volar to the critical line were classified as grade 0 (Fig. 3a); plates either touching or partially protruding from the critical line were classified as grade 1 (Fig. 3b); plates directly on or beyond the volar rim were classified as grade 2 (Fig. 3c). We quantified the amount of plate prominence by measuring the distance between the plate edge and the critical line (PCL) according to Kitay et al. [6]. Posteroanterior radiographs were also investigated to clarify plate placement for radioulnar direction and distal border. Radial or ulnar plate position was judged by measuring the distance between the ulnar corner of the plate and radius ulnar border (plate-to-radius ulnar border distance, PRU distance) (Fig. 4). To determine the distal limit of the plate in the posteroanterior radiographs, the distance between the plate end and distal radius (plate-to-distal radius distance, PDR distance) was measured. All measurements were adjusted using the radiolucent scale on the radiographs. The first author measured the radiographic parameters using a Digital Imaging and Communications in Medicine (DICOM) viewer (Yakami DICOM Tools, Kyoto University, Kyoto, Japan). The mean value of two measurements was used as the final value.

Pearson’s test was used for correlations between PCL distance and PDR distance. *p*-Value < 0.05 was considered statistically significant.

## Results

Lateral radiographs demonstrated grade 0, 1 and 2 prominences in 23 (63.9%), 9 (25.0%) and 4 (11.1%) plate–cadaver combinations, respectively, according to the Soong grading system. In every six plates, at least one radiograph showed more than a grade 1 prominence, whereas Acu-Loc2, HYBRIX and MODE showed grade 2 prominence (Table 1).

**Table 1:** Soong grade and PCL distance

	Soong grade			PCL distance (mm)		
	Grade 0	Grade 1	Grade 2	Mean	Max.	Min.
Acu-Loc2	2	2	2	1.12 ± 1.07	2.32	− 0.55
HYBRIX	3	2	1	0.06 ± 1.02	1.51	− 1.50
MODE	4	1	1	− 0.26 ± 1.11	1.33	− 1.51
VALCP	5	1	0	− 0.53 ± 0.69	0.87	− 1.26
VariAx	4	2	0	− 0.47 ± 0.78	0.57	− 1.78
DVR	5	1	0	− 0.77 ± 0.88	0.71	− 2.01
Total	23	9	4			

The critical line is a line drawn parallel to the volar cortex of the radial shaft, touching the most volar tip. All plates indicating more than grade 1 prominence

*PCL distance* plate-to-critical line distance, *Mean* mean value, *Max.* maximum value, *Min.* minimum value, *±* standard deviation

**Fig. 5:**
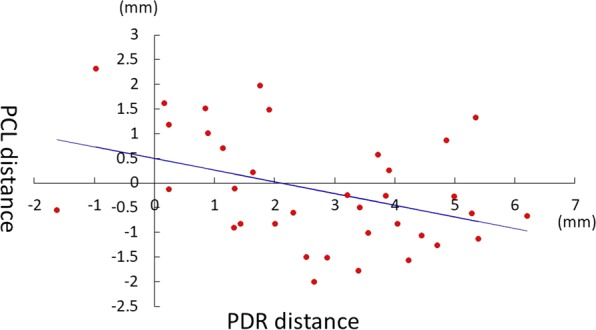
Plate-to-distal radius (PDR) distance was negatively correlated with plate to critical line (PCL) distance

Mean PCL distance was −0.77 to 1.12 mm, and four plates showed a negative value, except for Acu-Loc2 and HYBRIX. The maximum value of the PCL distance was 0.57 to 2.32 mm (Table 1). The maximum value of the PCL distance for VALCP, DVR and VariAx was less than 0.9 mm, whereas that for Acu-Loc2, HYBRIX and MODE was more than 1.3 mm. The mean PRU distance is presented in Table 2. No plate was positioned beyond the ulnar margin of the distal radius. The mean PDR distance is presented in Table 2. PDR distance was negatively correlated with PCL distance (*R* = − 0.40, *p* = 0.016) (Fig. 5).

**Table 2:** Mean PDR distance and PRU distance

	PDR distance (mm)			PRU distance (mm)		
	Mean	Max.	Min.	Mean	Max.	Min.
Acu-Loc2	0.24 ± 1.29	1.91	− 1.62	4.80 ± 1.77	6.88	1.24
HYBRIX	1.83 ± 1.08	3.90	0.84	4.77 ± 2.36	8.70	1.23
MODE	3.37 ± 1.81	5.39	0.24	3.03 ± 1.83	5.66	0.75
VALCP	4.20 ± 1.30	6.20	2.14	6.38 ± 1.99	8.31	2.20
VariAx	3.30 ± 0.85	4.45	1.63	3.59 ± 0.70	4.63	2.58
DVR	3.27 ± 1.66	5.28	1.14	4.91 ± 1.61	7.29	2.96

*PRU distance* plate-to-radius ulnar border distance, *PDR distance* plate-to-distal radius distance

## Discussion

We investigated radiographs of cadaver wrists with various VLPs placed in the most distal position without FPL tendon contact. The findings revealed that VLPs placed in this position may exhibit plate prominence on lateral radiographs. Moreover, the amount of protrusion varied depending on the plate used.

The FPL runs close to the volar ridge of the distal radius, which is known as the watershed line [9]. This bony landmark is regarded as important, because placement of the VLP beyond the watershed line can result in FPL rupture [7]. Soong et al. reported that grade 1 prominence was associated with an FPL rupture rate of almost 2%, whereas in case of grade 2 prominence, the rupture rate exceeded 4% [7]. Moreover, many clinical reports have used the volar ridge on lateral radiographs as an indicator of plate position [9, 11]. Limthongthang et al. conducted an anatomical study to investigate the Soong grade on lateral radiographs using commercial plates and cadavers [15]. In their study, plates were placed on the distal radius within the watershed line, then lateral radiographs were obtained. They reported that 90% of the lateral radiographs showed Soong grade 0, whereas the other 10% showed grade 1 [15]. To date, clinical as well as anatomical studies investigating lateral radiographs following VLP surgery have focussed on the watershed line and volar ridge. However, they have not been able to investigate FPL tendon contact with the implant. In this study, implant protrusion beyond the volar ridge was found in 36% of radiographs, even though the plate did not touch the FPL. This can be explained by the anatomical feature of the distal radius and related situation of the FPL tendon. The distal end of the volar radius is divided into two parts, viz. the medial and lateral columns [10]. The medial part is formed by a clearly palpable bony prominence, confirmed as the volar ridge on lateral radiographs [16], while the lateral half is formed by the lower prominence of the medial part. At the middle, a shallow groove is formed by the medial and lateral prominences [10]. The FPL runs in the vicinity of this groove, which is not a volar summit confirmed on lateral radiographs. Therefore, plate protrusion projecting over the volar ridge does not always indicate direct contact with the FPL tendon but does suggest a relative risk for irritation.

Kitay et al. recommended implant removal for symptomatic patients with plate positions within 3 mm of the volar ridge and suggested the necessity of implant removal when the PCL distance was more than 2.0 mm [6]. In this study, VALCP, DVR and VariAx showed PCL distance of less than 0.9 mm and Soong grade 0 or 1. When a surgeon uses these plates and lateral radiographs show Soong grade 2, the patient would be at risk for FPL irritation. Other implants, such as Acu-Loc2, HYBRIX and MODE, showed PCL distance of more than 1.3 mm. Plate prominence on lateral radiographs varied by plate selection. Nowadays, different widths or profiles of VLPs are in use. Thus, investigation of the PCL distance of various plates would be meaningful.

PDR distance showed a negative correlation with PCL distance, thus distal placement of the plate is a factor for higher Soong grade. The Soong grading system reflects the clinical outcome and has helped many surgeons avoid FPL rupture, although risk-free patients with Soong grade 1 or 2 surely exist. Estimation of the risk of FPL rupture using lateral radiographs alone would be insufficient. An additional indicator is necessary for deciding implant removal to avoid FPL rupture following VLP surgery [17]. Given advances in electronic devices, clinicians can now evaluate tendon status using ultrasound following VLP surgery [18]. Yamazaki et al. reported a risk assessment for tendon rupture after VLP surgery using audible crepitus [19]. They concluded that crepitus and volar placement of the implant were risk factors for tendon attrition after VLP surgery. More accurate estimation of the risk of FPL rupture would be possible if clinicians include ultrasound or audible crepitus among their assessment methods in patients who have undergone VLP surgery.

This study has several limitations. First, the study had a small sample size of plates and specimens, and the screw hole used multiple times led to screw loosening. To address this, two or more cortical screws were used if necessary. We prepared nine cadavers, and each plate was assigned to six cadavers to reduce deterioration of the cortical bone strength. Nevertheless, the cadavers were used several times; therefore, the order of plate placement might have affected the fixing force of the screw, associated with screw loosening. Second, we used formalin-fixed cadavers, which might alter the situation of muscles and soft tissues. Moreover, bone strength would decline compared with normal bone. Also, it was not possible to assess dynamic problems such as rubbing of the tendon over the plate. Matityahu et al. studied the contact pressure between fresh-frozen cadavers and VLP using a pressure sensor [20]. In their study, the contact pressure between the FPL and plate showed a 7% increase on wrist extension from 25° to 60°. Using fresh cadavers as well as different measurement instruments might increase the reliability of the present study. Third, we excised all soft tissues around the FPL, because it was necessary to confirm the relation between the FPL tendon and plate. This would lead to a change in the position of the FPL. The circumstances might differ in a normal living body. Nanno et al. investigated FPL movement using ultrasound in healthy volunteers. They reported that FPL moved ulnodorsally at the wrist dorsal flexion position during finger motion and ulnopalmarly at the wrist palmar flexion position with all five fingers in full extension [21]. Similarly, Schlickum et al. conducted an ultrasound investigation of patients with distal radius fracture following VLP surgery, comparing the FPL position in 0° wrist position and fingers extended with wrist held at 45° dorsal extension and with actively flexed index to little fingers. In their report, the FPL moved ulnarly in two-thirds of patients and radially in one-third of patients during wrist movement from 0° to 45° [22]. Fourth, all specimens had normal conditions with no fracture of the wrist. Patients with loss of reduction or inadequate reduction may have different results. Wurtzel et al. investigated plate and FPL tendon contact using cadavers and electric circuits, reporting that loss of volar tilt increased contact between the plate and FPL [23]. Fifth, although we placed the plate with the best fit in the radial–ulnar position, the plate may not be placed exactly straight because of individual differences. This problem could affect the measurements. Finally, we analysed plate prominence on radiographs, which might be affected by forearm rotation. To address this error, we used fluoroscopy and took radiographs a couple of times. The strength of this study is the ability to verify the radiograph of the VLP placed without FPL contact, which is usually difficult to investigate in the clinical setting.

In conclusion, VLP placed in the most distal position without FPL tendon contact can be identified as plate prominence on lateral radiographs. Implant protrusion was observed in 36% of plate–cadaver combinations, even if the plate did not touch the FPL. Acu-Loc2, HYBRIX and MODE can be placed more distally without FPL irritation than VALCP, DVR and VariAx. As VLP fixation for distal radius fracture becomes increasingly common, it is important to investigate whether risk-free protrusion or plate removal is necessary. Our data could provide surgeons with accurate knowledge regarding implant prominence following VLP surgery and will help them decide whether implant removal is necessary in these patients.

## Data Availability

The datasets used and/or analysed during the current study are available from the corresponding author on reasonable request.

## Abbreviations

VLP: volar locking plate
DRF: distal radius fracture
FPL: flexor pollicis longus
PCL: plate edge and the critical line
PRU distance: plate-to-radius ulnar border distance
PDR distance: plate-to-distal radius distance
